# Network Connections That Evolve to Circumvent the Inverse Optics Problem

**DOI:** 10.1371/journal.pone.0060490

**Published:** 2013-03-26

**Authors:** Cherlyn Ng, Janani Sundararajan, Michael Hogan, Dale Purves

**Affiliations:** 1 Neuroscience and Behavioral Disorders Program, Duke-NUS Graduate Medical School Singapore, Singapore, Singapore; 2 Department of Neurobiology, Duke University Medical Center, Durham, North Carolina, United States of America; 3 Center for Cognitive Neuroscience, Duke University, Durham, North Carolina, United States of America; University of Sussex, United Kingdom

## Abstract

A fundamental problem in vision science is how useful perceptions and behaviors arise in the absence of information about the physical sources of retinal stimuli (the inverse optics problem). Psychophysical studies show that human observers contend with this problem by using the frequency of occurrence of stimulus patterns in cumulative experience to generate percepts. To begin to understand the neural mechanisms underlying this strategy, we examined the connectivity of simple neural networks evolved to respond according to the cumulative rank of stimulus luminance values. Evolved similarities with the connectivity of early level visual neurons suggests that biological visual circuitry uses the same mechanisms as a means of creating useful perceptions and behaviors without information about the real world.

## Introduction

**Figure 1:**
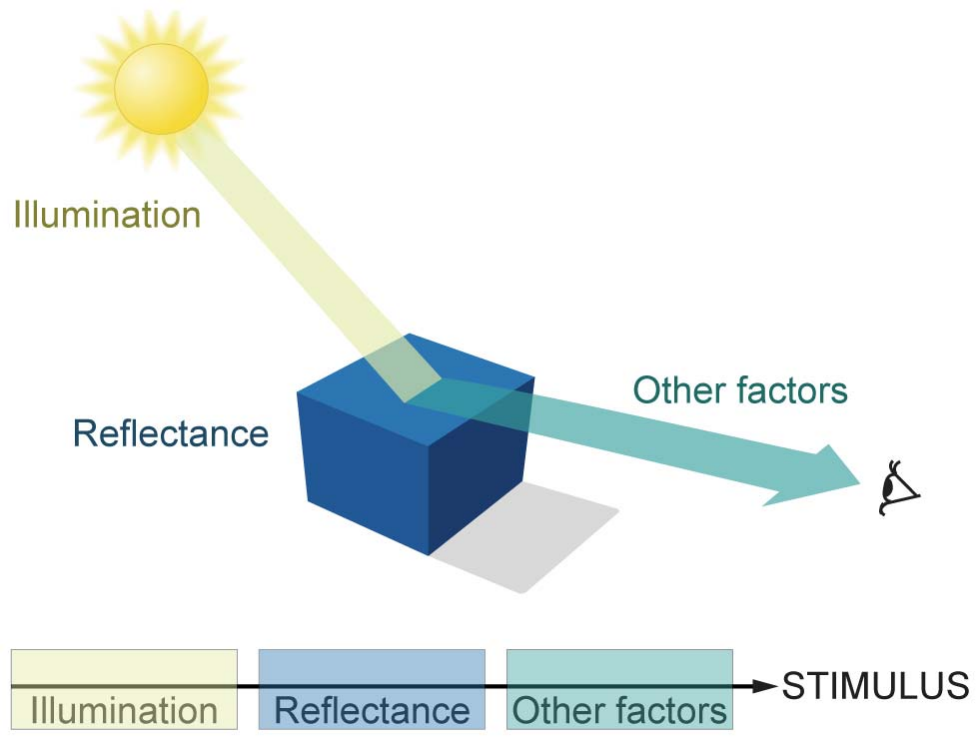
The inevitably uncertain meaning of luminance in visual stimuli (the inverse optics problem). Retinal luminance values are determined by combinations of illumination and reflectance, as well as a variety of other factors (e.g., atmospheric transmittance, spectral content, occlusion, object distance, etc.). These physical determinants of retinal luminance values are conflated in visual stimuli and cannot be disentangled by any algorithmic process (adapted from [20], pp.22).

Perceptions of lightness and brightness elicited by stimulus luminance are the basis of all visually guided behavior. Light projected onto the retina, however, is unable to specify the generative sources of luminance in the world in which we and other visual animals behave [1]. As shown in Figure 1, no logical operation on retinal luminance values can retrieve the surface reflectance properties of objects, their illumination, or any other relevant physical factors. As a result, it is difficult to understand how visual circuitry generates useful perceptual and behavioral responses.

To appreciate the problem that the conflation of reflectance and illumination presents, imagine a range of objects with different physical compositions in the complex illumination that occurs in natural circumstances. The same object surfaces would often be in different illumination, and thus return different luminance values to the observer. Conversely, the luminance returned from two physically different surfaces under different illuminants would often be the same. It would thus be of little use to respond to the retinal luminance as such. Indeed numerous studies have shown that the visual system does not represent absolute luminance in percepts [2]–[5]. Since visually guided behavior is generally successful despite the inaccessibility of real world source properties, these facts raise the question of how visual processing accomplishes this feat.

One way of addressing this question is based on efficient neural coding, a term used to describe models of visual processing that minimize energy expenditure while maximizing the transfer of information available in natural scenes [6]–[18]. A corollary is that visual information will be transmitted more efficiently if the luminance values and their higher order statistics are made independent. Predictive coding, sparse coding, principle component analysis, whitening and independent component analysis have been proposed as models of biological visual processing. A validation of efficient coding is that it can account for some aspects of the receptive fields of early level visual neurons [9]–[17].

Although these models demonstrate the advantages of efficient information transfer in visual processing, they do not explain: 1) how visual agents are behaviorally successful in the face of the inverse problem; 2) why the lightness and brightness values we see in response to stimulus luminance, as well as perceptions of color, form, distance and motion are not the physical parameters measured in the visual environment; and 3) how neural connectivity in biological visual systems instantiate perceptions that successfully guide behavior.

A different approach that addresses these questions supposes that visual connectivity and its perceptual consequences have emerged over evolutionary and individual time simply by associating patterns of retinal luminance and other stimulus parameters with behaviors that lead to reproductive success [1], [19]–[21]. In this conception, the primary goal of vision is not efficient coding (although efficiency is obviously important), but finding a way around the fact that information about the physical properties of world is not available. An indication of the strategy being used is that the perception of light intensity and other perceptual qualities accord with the cumulative ranks of the frequencies of occurrences of stimuli [1], [19]–[21]. The present study shows that simple neural networks evolved on this basis give rise to connectivity that is comparable to biological networks in early vision. These observations show that, in principle, vision can generate successful behavior without access to (or measurement of) the physical world.

## Results

### The Paradigm

**Figure 2:**
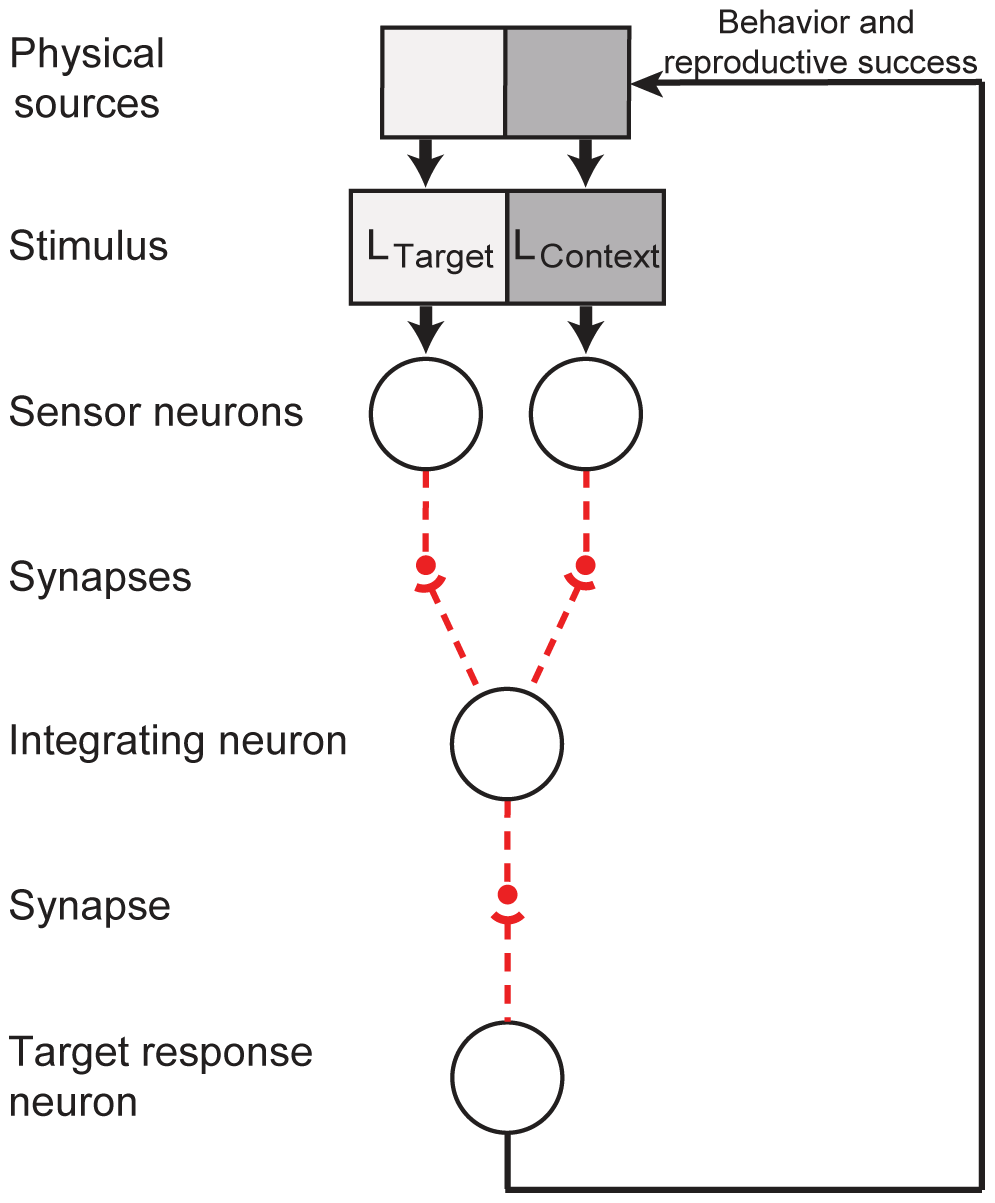
The network, its potential connectivity, and the evolutionary paradigm. Stimulus luminance values (L_Target_ and L_Context_) were received by two adjacent sensor neurons. The potential connectivity of the circuit (dashed red lines) was modeled according to basic principles of synaptic physiology (see Methods). The small filled circles indicate presynaptic terminals and the short arcs postsynaptic sites. The value at the response neuron indicates the output of the circuit to the luminance value at the target sensor given the context value, which in turn determined the relative reproductive success of each evolving circuit. The strength and sign of the connections that eventually evolved were based solely on their contributions to reproductive success.

To explore how vision might work on a wholly empirical basis, we used feed forward networks each comprising four neurons that could evolve full connectivity (Figure 2). Two neurons were adjacent sensors whose visual fields covered 1 square degree. The luminance values received by the sensors were forwarded via evolvable synaptic connections to an integrating neuron, and subsequently to a response (output) neuron. Although photoreceptors respond to log luminance levels [22], the networks here were presented with pairs of absolute luminance values to simulate naturally arising stimulus elements on the retina. The values at the output neuron indicated the circuit’s response to the luminance at the target (left) sensor given the luminance at the context (right) sensor. The terms target and context do not refer to a particular sensor as one sensor serves as context for the other. At the outset of evolution all 3 connections in the circuit existed only as possibilities that could–or could not–evolve non-zero positive or negative synaptic strengths.

The strength of each potential synapse in the evolving circuits accorded with the neurobiological facts that: 1) a postsynaptic conductance change, whether excitatory or inhibitory, is always positively correlated with presynaptic depolarization; 2) the synaptic transfer function is non-linear [23]; and 3) synapses can be either excitatory or inhibitory, but not both.

### Experience of the Evolving Circuits

**Figure 3:**
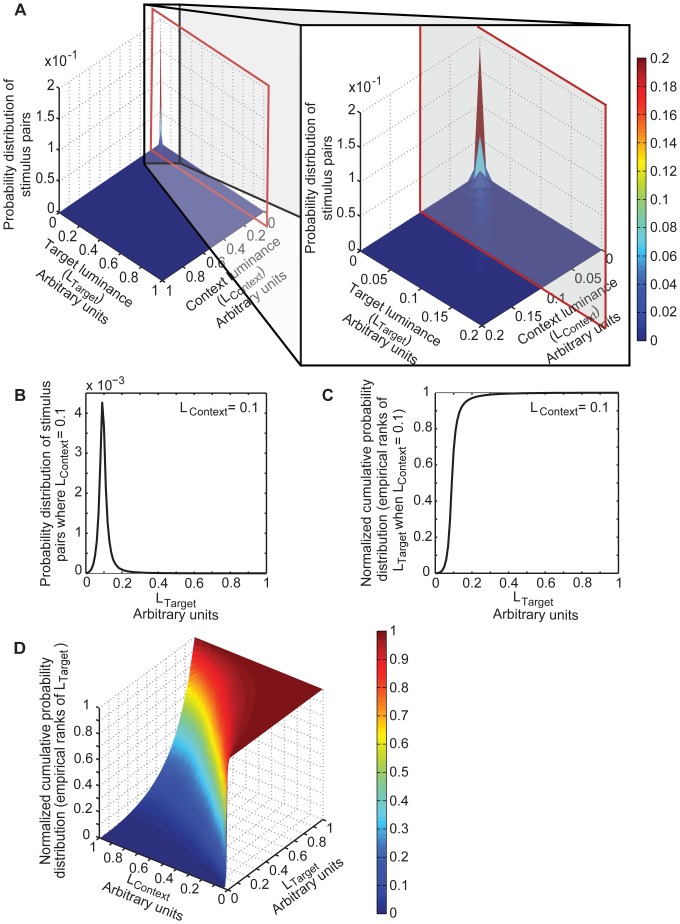
Experience of the evolving circuit population. (A) Probability distribution of different L_Target_ and L_Context_ luminance pairs derived by sampling real world images. Inset shows a magnification of the graph at lower luminance values. The section outlined in red indicates the probabilities of any L_Target_ value occurring with a particular L_Context_ value (0.1 in this example). (B) The probability distribution of the section in (A). (C) The cumulative probability of the distribution in (B) normalized with respect to the highest cumulative frequency of occurrence. (D) Normalized cumulative probability distributions for all L_Target_ values given different L_Context_ values.

Figure 3A shows the probability distribution of all possible stimulus luminance pairs that the evolving circuits could experience, drawn from a database of natural images (see Methods). A bias towards lower stimulus luminance values occurs because: 1) more combinations of reflectance and illumination are possible for lower luminance values than higher ones (Figure S1); and 2) there are more low reflectance objects under low illumination in natural scenes [24], [25]. A bias towards similar adjacent luminance values (the diagonal ridge in Figure 3A) occurs because nearby natural objects tend to be made of the same material and illuminated in the same way [26], [27]. A section through this topology (red outline) indicates the probabilities of stimulus luminance values at the target sensor, given a particular luminance value at the context sensor. Figure 3B shows the distribution of values in the section in Figure 3A, and Figure 3C represents this distribution as the accumulated experience of the evolving networks. Figure 3D shows the full range of accumulated experience for all possible target values given different context values, i.e. the cumulative probabilities of all sections parallel to the red outline through the topology in Figure 3A. Success, and thus a circuit’s chance of reproducing members of the next generation, was measured by comparing its responses to the empirically derived topology in Figure 3D (see Methods and Discussion).

### Evolution of the Networks

**Figure 4:**
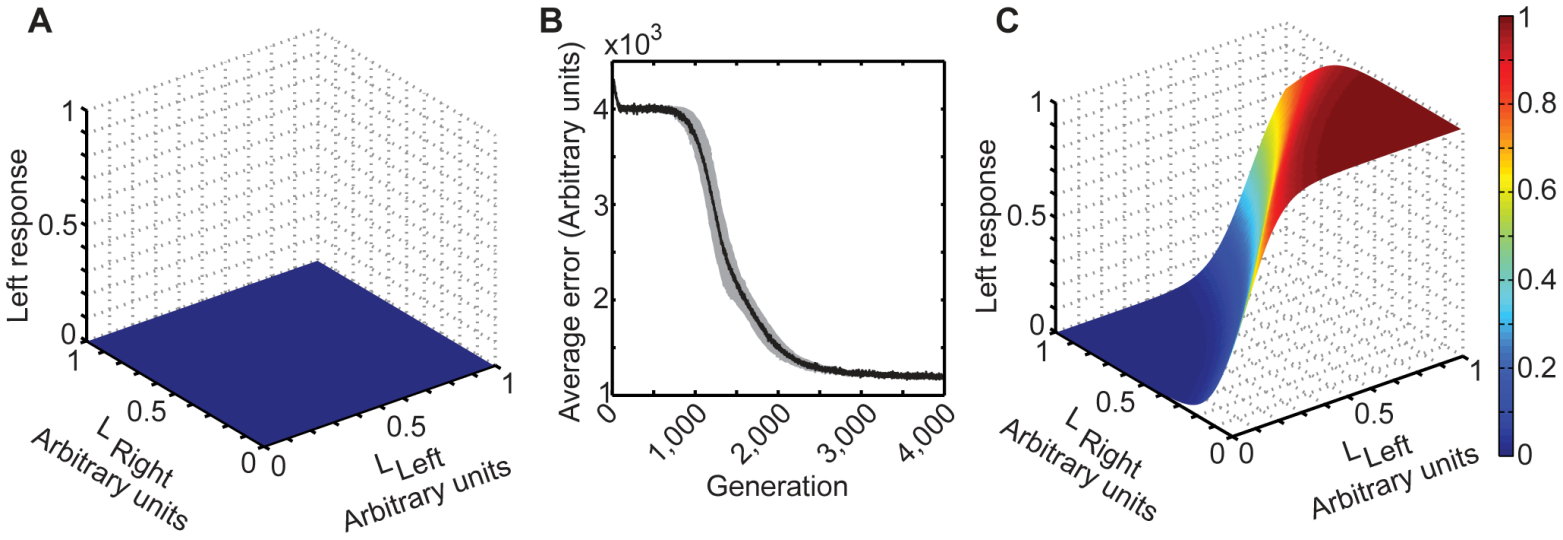
Evolution of circuit responses. (A) The average responses of the output neuron defined a nearly flat topology near 0 at the beginning of evolution. (B) The average evolution profile of 25 populations of 500 circuits over the course of 4000 generations; the standard deviation is indicated in gray. The rapid improvement over the first few generations arises because all connections are initialized near 0; once some connectivity evolves, the responses quickly attain a degree of success. (C) The average responses of the output neuron of the 25 populations in (B) at the end of evolution. The absolute standard deviation of the responses of the output neurons ranged between 0 and 0.02.

Beginning with responses arising from synaptic strengths randomly initialized near 0 (Figure 4A), we examined the average performance of populations of 500 networks in 25 different simulations. Performance over ∼2000 generations rapidly improved, and then began to asymptotically approach a plateau (Figure 4B). Figure 4C shows the average evolved responses for 25 simulations. Comparison of the topology in Figure 4C with that in Figure 3D indicates that the responses of the circuits evolved to approximate the cumulative experience of the target values in each stimulus, as expected. The only constraints on the evolving networks were the restrictions on the number of neurons in the network and the evolvable synaptic transfer function.

### Evaluation of Network Success

Given the rudimentary nature of the evolutionary paradigm, it was not a given that the networks would develop responses sufficiently similar to those evident in psychophysics to make meaningful comparisons with biological visual circuitry. We thus evaluated whether the evolved networks mimicked human perceptual functions elicited by similar stimuli.

**Figure 5:**
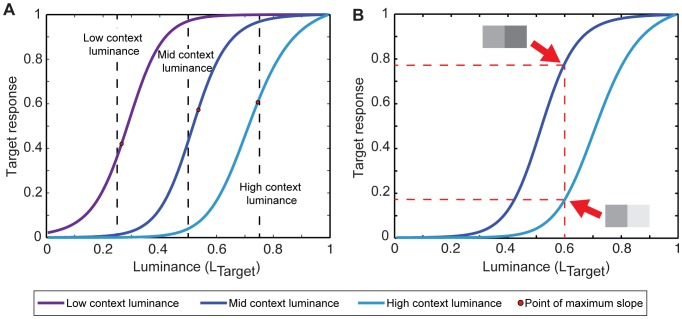
Comparison of networks’ responses with human psychophysics. (A) Magnitude estimation functions generated by the evolved circuits. Average circuit responses when the target sensor was presented with varying luminance values and the luminance at the context sensor was held constant at three different levels (dashed lines). The curves show the average circuit responses to increasing target luminance values in different contexts. (B) Circuit responses to stimulus contrast. The two curves are the responses of the target sensor to luminance values presented with a lesser (blue) or greater (turquoise) luminance at the context sensor. Dashed lines indicate different circuit responses to the same target luminance when presented in these different contexts.

The relevant psychophysics are magnitude estimation functions and contrast functions [5]. In magnitude estimation studies the psychophysical functions observed are perceptions of luminance in response to a range of target reflectance values presented on a constant background under steady illumination [3], [5], [19]. The phenomena evident in human magnitude estimation psychophysics were apparent in all 25 simulations of evolved circuit responses (Figure 5A) [19]. Thus: 1) the exponent for responses to target luminance values above the context luminance is a power function similar to Stevens’ Law. They increase with increasing background luminance (∼0.24 in low, ∼0.32 in mid and ∼0.57 in high luminance backgrounds); 2) the functions change direction when the context luminance is about the same as the target luminance; 3) when the target luminance values are presented with a higher context luminance, the functions are shifted to the right; and 4) the slopes of the functions are steepest when the target and background luminance values are similar (the “crispening effect”). This last effect also means that more frequently occurring luminance patterns are more finely resolved (and thus better discriminated) than less frequently occurring ones, as is characteristic of human performance [28].

Contrast refers to the relative lightness of two adjacent stimulus luminance values. In human psychophysics one luminance value (the “context”) affects the perception of the other (the “target”) [5], [20]. Thus the same target luminance value presented together with a darker context value is seen as lighter than when presented together with a lighter contextual luminance value. The circuit responses to contrast in all 25 simulations were again similar to those observed in human psychophysics (Figure 5B).

### Network Mechanisms

**Figure 6:**
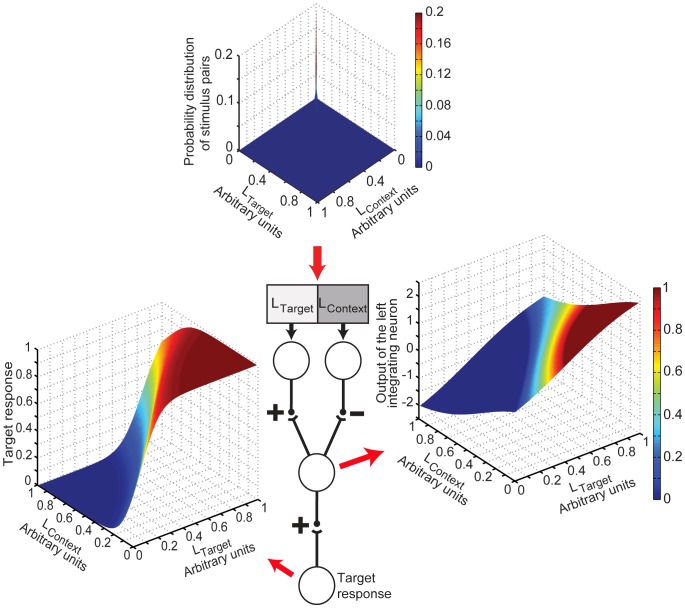
The evolved mechanisms used to generate successful responses. The central diagram shows the connectivity that consistently evolved beginning with the circuit in Figure 2; excitatory synapses are indicated by (+) and inhibitory synapses by (−). The experience of the evolved network (Figure 3D) is determined by the frequency of occurrence of luminance patterns (upper panel, redrawn from Figure 3A). The evolved connectivity from the sensors to the integrating neuron represents this experience as a weak topology (right panel). The evolved connectivity from the integrating to the output neuron transforms this topology into an approximation of the network’s cumulative experience (left panel). The evolved responses to particular stimuli are approximations of the percentile rank of all the stimulus luminance pairs in the networks’ experience (see Table 1 for average values of evolved parameters).

Given this similarity between the evolved network responses and human psychophysics, we next asked if the evolved connectivity might provide some insight into the principles of the vastly more complicated circuitry evident in biological systems. The central diagram in Figure 6 shows the organization that evolved in all 25 simulations starting with the potential circuitry in Figure 2; the networks were free to evolve any of the potential synaptic connections that had randomly assigned values at the beginning of the simulations. The evolved features, whose details are given in Table 1, were:

**Table 1:** Means and standard deviations of the evolved parameters (A, B, C; see Methods) of the circuit in Figure 6 over 25 simulations.

Synapse	Sign	A	B	C
Direct synapse to theintegrating neuron	+	3.4±0.07	3.4±0.10	1.4±0.05
Indirect synapse to theintegrating neuron	−	3.0±0.06	3.6±0.11	1.2±0.05
Synapse to theresponse neuron	+	1.0±0.01	5.8±0.12	0.6±0.24

1. The signs of the synapses linking the two sensors to the integrating neuron were always opposite.
2. The direct connection from the target sensor to the integrating neuron was always excitatory, and the indirect connection from the context always inhibitory.
3. The synaptic connection from the integrating neuron to the response neuron was always excitatory.

The evolved connectivity in Figure 6 thus uses opposing synaptic transfer functions between the sensor and integrating neuron, i.e. excitation from the target and inhibition from the context, to give responses that follow the same trends as the network’s cumulative experience (see Figure 3D). As luminance values at the target sensor neuron increase for a given context, the evolved values at the integrating neuron also increase. This is achieved by the excitatory evolved direct connection from the target sensor that increases the outputs at the integrating neuron as a function of luminance, scaling them along the L_Target_ axis in the right panel in Figure 6. At the same time, the inhibitory indirect connection to the integrating node decreases the outputs of the node as a function of the adjacent, “context” luminance values thereby scaling them along the orthogonal L_Context_ axis in the right panel in Figure 6.

The responses obtained by summing these direct and indirect influences at the integrating neuron, however, do not correspond to the circuit’s accumulated experience with the stimulus luminance pairs (compare the topology in the right panel in Figure 6 with Figure 3D). In particular, the values at the integrating neuron are negative when the target luminance value is minimal and the contextual value maximal, and positive in the opposite case. The output values of the integrating neuron are therefore transformed by a further excitatory synapse such that they effectively track the topography of accumulated experience in Figure 3D (the left panel in Figure 6) and allow non-linear responses. This synapse must be excitatory so that values at the response node are always positive; negative frequencies of occurrence and/or negative accumulated experiences are not meaningful. Finally, the combination of the evolved synaptic signs in Figure 6 means that the synapses in the circuit all have the positive transfer function evident in biology, i.e., a direct relationship between presynaptic depolarization and neurotransmitter release. Any other arrangement of connections would require at least one synapse to reduce transmitter release when depolarized.

Given these mechanisms, magnitude estimation effects arise from the excitatory direct connection in the evolved networks, which increases responses as the luminance values increase in the target sensor. Contrast arises from the inhibitory indirect connection that progressively decreases the responses as the luminance of the context increases. Thus a given target luminance value should be seen as lighter in low luminance contexts, and become progressively darker as the context luminance increases, as is the case in human perception [1]. In short, contrast effects are the result of the lateral inhibition that is needed to rank environmental luminance values in the full range of each network’s experience. As the cumulative ranks of luminance are influenced by both their values and the correlations among adjacent pixels, these aspects of the natural images are needed to produce the target/context opponent mechanism.

### Light Adaptation

In addition to circumventing the inverse problem (see Figure 1), the evolved circuitry in Figure 6 has another important advantage: the mechanism ensures that the relative sensitivity of the network responses is reset by the frequencies of occurrence of the luminance values in a stimulus. Just as biological visual systems use center-surround receptive field organization to adjust the rates of neuronal firing to accord with ambient light [29], lateral inhibition in the evolved networks adjusts the responses according to the frequencies of occurrence of luminance values in a particular context (see Figure 5). The sensitivity of the output neuron is thus maximal around the context value, where stimuli in this range occur most frequently. The evolved circuit in Figure 6 thus automatically adjusts the response to the target luminance in accord with the luminance of the context.

## Discussion

### Operation of Circuitry Evolved on a Wholly Empirical Basis

Given the inability of photosensors to specify underlying physical sources of light coming from the environment, one way–we would argue the only way–a visual animal can relate visual stimuli to useful responses is to evolve neural connectivity that bridges the objective and subjective domains by trial and error. In this conception of vision, the circuit response to a stimulus is reflexive in the sense that there is no feature detection, image analysis or feature representation. All the work that these postulated processes entail has been done over evolutionary time by trial and error that determines, by natural selection, the circuitry that visual animals are born with. There is of course modification of visual and other neural circuitry over an individual’s lifetime, but the effects of neural plasticity only modulate inherited connectivity [30], [31].

### Relevance to Biological Circuitry

Although the artificial neural circuits we evolved are vastly simpler than the connectivity of even the most primitive biological visual system, given that they confront the same problems in a similar environment, the principles they exhibit should be broadly similar to those underlying animal vision. In fact, the organization of the evolved circuits in Figure 6 suggests an empirical rationale for some key aspects of the connectivity evident in biological visual systems. The most obvious of these is lateral inhibition, a feature found in all animal visual systems that has generally been considered a way to “sharpen” visual and other sensory representations [32], [33]. The present results suggest that the prevalence of lateral inhibition at the early stages of biological visual processing arises because it is essential to produce the needed perceptual responses to any particular stimulus within an agent’s experience (see Figure 6 and above). Whether luminance intensity is directly correlated with transmitter release (as it is here and in invertebrate photoreceptors) or inversely correlated (as it is in vertebrate photoreceptors), lateral inhibition is essential for successful responses in the above environment and emerges as a consequence of the decrease in occurrence of higher luminance values.

Another issue is the implied receptive field properties of neurons in the evolved circuits in Figure 6. The emergence of lateral inhibition suggests that if we were to evolve arrays that receive and respond to larger areas of the visual space, the connectivity of the circuits would lead to integrating neurons with opposing center-surround receptive fields, as in the retina, thalamus and input layer of the primary visual cortex [32], [34], [35].

Most important, cumulatively ranking luminance values gives rise to non-linear network responses that accord with human psychophysics. The connectivity also results in lateral inhibition, is similar to the physiology underlying light adaptation, and is automatically efficient [8].

### Conclusions

A central problem facing biological visual systems–or artificial neural circuits responding to projected light–is that the physical properties underlying stimulus luminance values are not available in images. Much evidence now suggests that biological vision deals with this quandary by generating perceptions and behavior empirically. The present study explores underlying circuit mechanisms that could accomplish this goal. The results show that: 1) simple circuits can evolve connectivity and responses that circumvent the inverse problem by cumulatively ranking stimulus luminance values in the full range of accumulated experience; 2) the responses generated in this way accord with the human psychophysical responses to luminance; 3) the evolved mechanisms that accomplish this goal can be understood in neurobiological terms; 4) the mechanisms have been determined entirely by the agents’ history and are thus reflexive rather than analytical; and 5) the mechanisms of the evolved connectivity suggest rationales for lateral inhibition, opposing receptive field organization, and adaptation to ambient light levels. In sum, circuitry evolved on a wholly empirical basis can generate behavioral success in a physical world whose features cannot be apprehended, providing a different way of understanding the organization and purposes of biological visual systems.

## Materials and Methods

### Determination of the Frequency of Occurrence of Stimuli

The frequency of occurrence of a given luminance pair falling on the two adjacent circuit sensors in Figure 2 was determined by extraction with a corresponding template repeatedly applied to 4,167 natural images [13], [20]. Each square degree in the images comprised 3600 pixels (60×60); the template was thus 60×120 pixels (adjacent 1 deg^2^ patches). The template was moved horizontally and vertically over each image in 10 pixel increments, resulting in ∼53 million samples. The luminance at each sensor was taken as the mean luminance of each square degree. The probabilities of occurrence of luminance values determined in this way were similar to those in previous reports.

The images in the database provided a calibrated luminance value at each pixel ranging from 0 to 70000 cd/m^2^. Because 92% of the luminance values in the database fell below 3000 cd/m^2^, values from 3000 to 70000 were too sparse to sample, leaving ∼49 million potential stimuli for presentation to the networks. These values were normalized to range between 0 and 1. In each generation, 10,000 stimuli were drawn at random from this set and presented to the circuits. Data extraction from natural images was done in Matlab (R2009a, The MathWorks, Natick, Massachusetts, USA).

### Synaptic Transfer Function

The synaptic transfer function we used can be formally expressed as
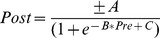
where *Post* is the magnitude and sign of the postsynaptic conductance change, and *Pre* is the presynaptic membrane potential. A determines the sign of a synapse (excitatory or inhibitory), and B and C control the range of input values a given neuron could respond to. The synaptic strength –the magnitude of synaptic effect on the postsynaptic cell–is determined by all three of these factors. A was randomly initialized to 0.01 or −0.01, B to 0.01 and C to 0. Thus at the outset of evolution all *Post* values were near 0 with randomly assigned signs.

### Performance and Circuit Reproduction

Populations of 500 circuits reproduced based on their individual lifetime performances, i.e. the summed success of their responses to 10,000 stimulus pairs. Performance error was measured as the sum of the absolute difference between each circuit’s responses and the percentile ranks of the respective stimuli in the circuit’s lifetime experience:
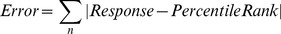
where n is the total number of stimulus pairs (10,000) in the circuit’s lifetime experience.

The fitness of the k^th^ circuit is its total lifetime error normalized to the maximum error among all circuits of the population (*max.Error*) in that generation and expressed in arbitrary units as:
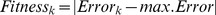



A roulette wheel with 500 radial sectors determined a circuit’s probability of reproduction, each sector’s arc corresponding to the fitness of a circuit in the population [36]:
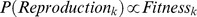



The wheel was spun 500 times and the circuit identified on each spin was added to the reproduction pool. This method in which any given circuit could be selected multiple times, means that more successful circuits reproduced often, but also that less successful ones could reproduce occasionally.

Other computational techniques (e.g., back propagation) could also have been used to generate successful neural networks. We chose an evolutionary algorithm because it is biologically feasible [37]. Although evolution does not conform to any mathematical model, in computational terms it can be thought of as a process that minimizes the errors an agent makes in responding to the environment over species and individual history.

### Novelty

To generate the novelty essential to any evolutionary process, diversity was introduced in two ways. First, each circuit from the reproduction pool was given an 80% chance of interchanging its connectivity with another. When a pair of circuits was chosen for hybridization, the properties of the synapses in each circuit were grouped, listed and exchanged at a random site (Figure S2). To introduce diversity in a way that allowed smaller changes, each circuit was also given a 20% chance of having a small value added to each of its evolving synaptic parameters (see above). The mutation values of A, B, and C were randomly chosen from a Gaussian distribution with a mean of 0 and standard deviation 0.01, and summed with the pre-existing value, thus changing the evolving values of the synaptic transfer function by small amounts [37]. A and C were allowed to assume any sign while B was constrained to be non-negative (see Results for explanation).

## Supporting Information

Figure S1 **Explanation of the bias towards low luminance values at any level of ambient light.** The filled circles along each of the 10 iso-luminance curves represent possible reflectance (R) and illumination (I) combinations underlying stimulus luminance values (the R and I values range from 0 to 1 in arbitrary units, and have uniform marginal distributions). Note that more RI combinations are possible for lower luminance values (the longer iso-luminance curves) than higher ones (the shorter curves), thus increasing the probability of co-occurring lower values.
(TIF)

Figure S2 **Hybridization.** The top panel shows two networks selected for hybridization. The point of hybridization was randomly chosen (indicated by the red arrows). The bottom panel shows the progeny after exchanging connections about the chosen point.
(TIF)
