# Supplementary figures and images for: Network Connections That Evolve to Circumvent the Inverse Optics Problem
Source: PLoS One. 2013 Mar 26;8(3):e60490. doi: 10.1371/journal.pone.0060490 (PMC3608599; doi:10.1371/journal.pone.0060490)

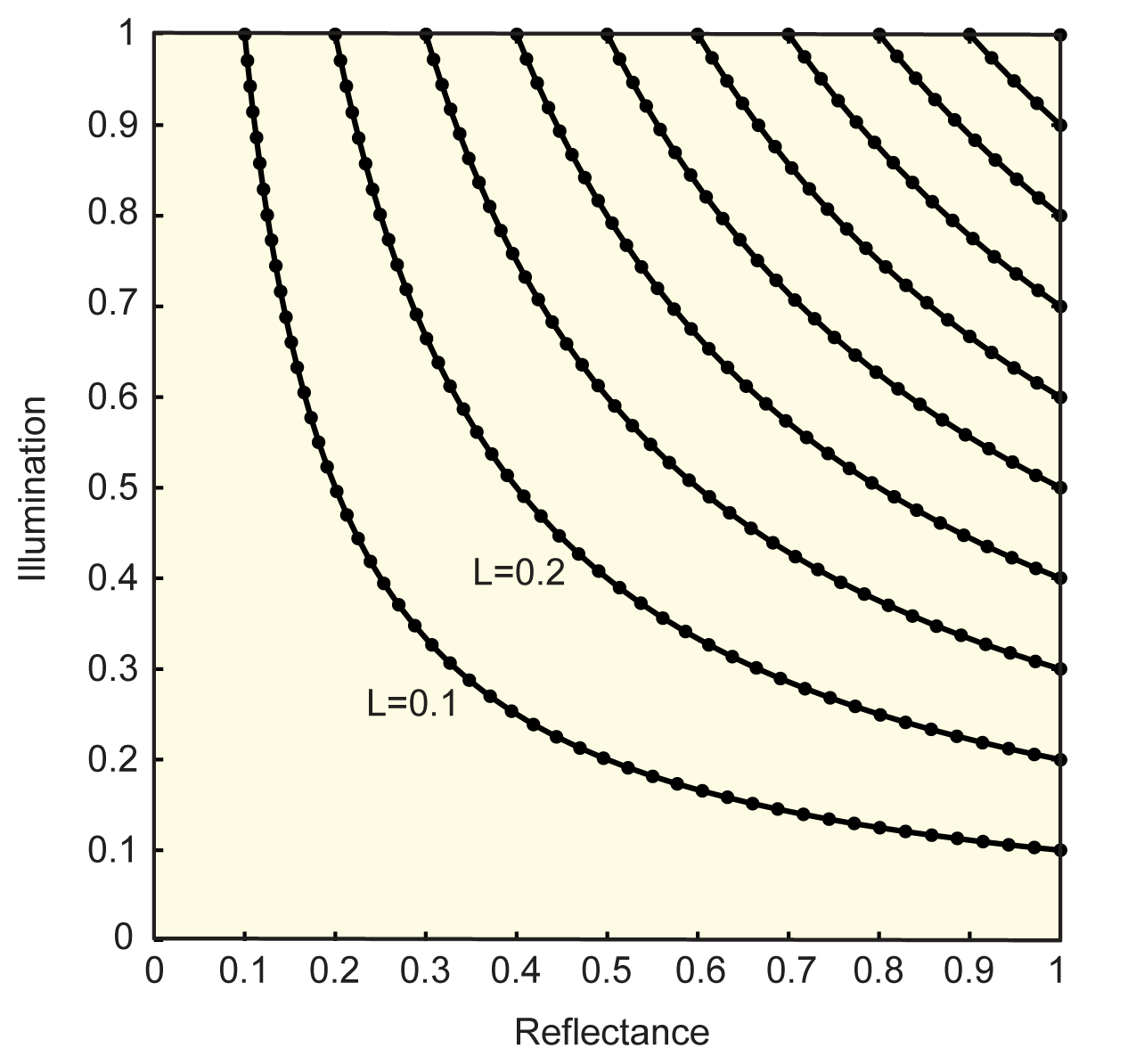

Supplement: Figure S1 — Explanation of the bias towards low luminance values at any level of ambient light. The filled circles along each of the 10 iso-luminance curves represent possible reflectance (R) and illumination (I) combinations underlying stimulus luminance values (the R and I values range from 0 to 1 in arbitrary units, and have uniform marginal distributions). Note that more RI combinations are possible for lower luminance values (the longer iso-luminance curves) than higher ones (the shorter curves), thus increasing the probability of co-occurring lower values. (TIF) [file pone.0060490.s001.tif]

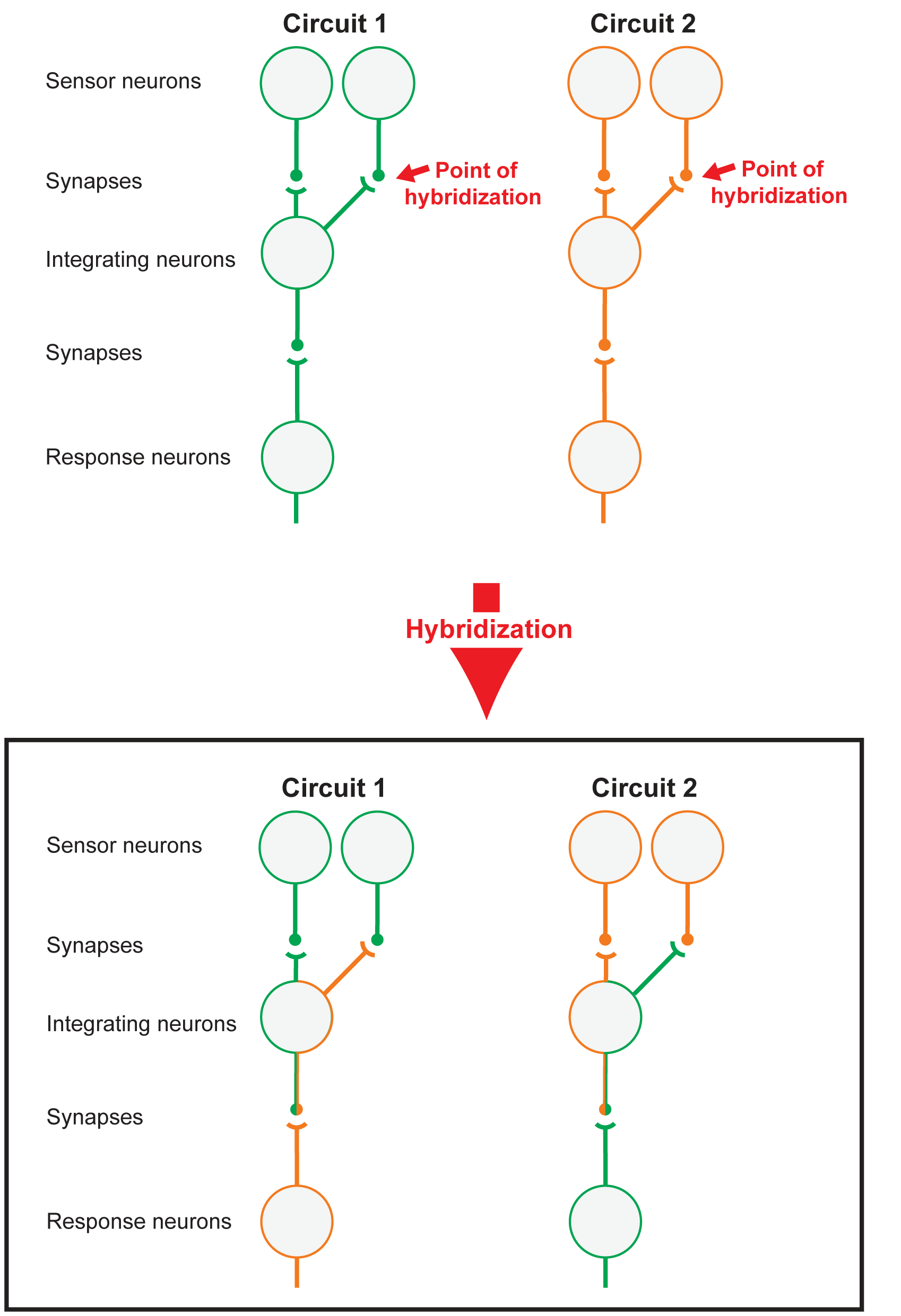

Supplement: Figure S2 — Hybridization. The top panel shows two networks selected for hybridization. The point of hybridization was randomly chosen (indicated by the red arrows). The bottom panel shows the progeny after exchanging connections about the chosen point. (TIF) [file pone.0060490.s002.tif]
